# Porcine Parvovirus 7: Evolutionary Dynamics and Identification of Epitopes toward Vaccine Design

**DOI:** 10.3390/vaccines8030359

**Published:** 2020-07-05

**Authors:** Dongliang Wang, Jinhui Mai, Yi Yang, Naidong Wang

**Affiliations:** Hunan Provincial Key Laboratory of Protein Engineering in Animal Vaccines, Laboratory of Functional Proteomics (LFP), Research Center of Reverse Vaccinology (RCRV), College of Veterinary Medicine, Hunan Agricultural University, Changsha 410128, China; dongliangwang@stu.hunau.edu.cn (D.W.); jinhuimai@stu.hunau.edu.cn (J.M.); yiyang@hunau.edu.cn (Y.Y.)

**Keywords:** PPV7, Cap, B cell and T cell epitopes, vaccine, evolution

## Abstract

Porcine parvovirus 7 (PPV7) belonging to the genus *Chapparvovirus* in the family *Parvoviridae*, has been identified in the USA, Sweden, Poland, China, South Korea and Brazil. Our objective was to determine the phylogeny, estimate the time of origin and evolutionary dynamics of PPV7, and use computer-based immune-informatics to assess potential epitopes of its Cap, the main antigenic viral protein, for vaccines or serology. Regarding evolutionary dynamics, PPV7 had 2 major clades, both of which possibly had a common ancestor in 2004. Furthermore, PPV7 strains from China were the most likely ancestral strains. The nucleotide substitution rates of *NS1* and *Cap* genes were 8.01 × 10^−4^ and 2.19 × 10^−3^ per site per year, respectively, which were higher than those reported for PPV1-4. The antigenic profiles of PPV7 Cap were revealed and there were indications that PPV7 used antigenic shift to escape from the host’s immune surveillance. Linear B cell epitopes and CD8 T cell epitopes of Cap with good antigenic potential were identified in silico; these conserved B cell epitopes may be candidates for the PPV7 vaccine or for the development of serological diagnostic methods.

## 1. Introduction

The family *Parvoviridae* contains two subfamilies: *Parvovirinae* and *Densovirinae*, whose hosts are vertebrates and arthropods, respectively [1]. The subfamily *Parvovirinae* is further divided into nine genera: *Tetraparvovirus*, *Copiparvovirus*, *Erythroparvovirus*, *Dependoparvovirus*, *Aveparvovirus*, *Bocaparvovirus*, *Amdoparvovirus*, *Protoparvovirus* and *Chapparvovirus* [1,2]. To date, seven genotypes of porcine parvoviruses (PPV) have been discovered in pigs, and belong to four genera: *Protoparvovirus* (PPV1), *Tetraparvovirus* (PPV2–3), *Copiparvovirus* (PPV4-6) and *Chapparvovirus* (PPV7), based on the similarity of non-structural protein 1 (NS1) [2,3]. PPV1, first identified in 1965 in Germany [4], is a major cause of reproductive failure in pigs, characterized by mummified fetuses, infertility, early embryonic death, stillbirths and delayed returns to the estrus [5,6]. Subsequently, six other PPV genotypes (PPV2–PPV7) were identified using novel techniques, including next-generation sequencing [2,7,8,9,10,11].

In 2016, PPV7 was first identified by the metagenomic sequencing of rectal swab samples from pigs in the USA [2]. Subsequently, PPV7 infections were reported in Sweden, Poland, China, South Korea and Brazil [12,13,14,15,16,17,18,19]. PPV7 is a small, single-stranded linear and non-enveloped DNA virus with a genome of ~4 kb that contains two major open reading frames (ORFs): ORF1 encodes non-structural protein 1 (NS1) responsible for viral replication, and ORF2 encodes the major structural capsid protein (Cap) or VP2 protein [2]. PPV Cap is also the major antigenic component, with an important role in eliciting neutralizing antibodies against viral infection [20]. In addition, it is a prospective antigen for subunit vaccine design and serological diagnosis. A vaccine based on the Cap could elicit antibodies to neutralize virus infection by blocking virus entry [21]. Thus, the Cap could be regarded as an effective antigenic component for PPV7 vaccine design and development. Currently, there is no, or very limited, information about its antigenic structure and immunogenic profiles, thus, the immune profiles of PPV7 Cap are required to develop PPV7 Cap-based vaccines against PPV7 infection.

Despite limited knowledge regarding PPV7 pathogenicity, its presence in aborted pig fetuses suggests that it causes reproductive failure [17]. Co-infections are more frequent than single infection in swine herd; multiple infectious pathogens such as PCV2 and PPV can impact the outcome of respiratory infections and deserve [22]. In addition, co-infections with PPV7 and PCV2 were recently reported [23], although whether this co-infection enhanced the severity of porcine circovirus associated diseases (PCVADs) has yet to be determined. To better understand the molecular evolution and genetic diversity of this newly emerging PPV7, we analyzed the phylogeny and estimated the time of origin and evolutionary dynamics of PPV7. Importantly, computer-based immune-informatics were used to assess the potential B cell and CD8 T cell epitopes of the PPV7 Cap, which will greatly facilitate the development of new generation vaccines against PPV7 infection and contribute to the development of effective serological diagnostic methods.

## 2. Materials and Methods

### 2.1. Sequence Datasets

A total of 45 complete (or partial) genomes, and 59 NS1 and Cap complete coding sequences, were collected from GenBank to perform phylogenetic analysis. Detailed information of PPV7 and reference *Parvoviridae* sequences are summarized in Tables S1 and S2, respectively.

### 2.2. Multiple Sequence Alignment

NS1 and Cap amino acid sequences were derived from 59 PPV7 genomes. Sequence identity was analyzed and aligned with the Clustal W method of the MegAlign program of DNASTAR, version 7.10 (Lasergene) (DNASTAR, Inc., Wisconsin, USA). Amino acid sequences were aligned with the ESPript 3.0 online tool (ESPript 3.0, http://espript.ibcp.fr/ESPript/ESPript/, 4 July 2020).

### 2.3. Phylogenetic and Evolution Dynamic Analysis

Sequences were aligned with Clustal W, implemented in MEGA 7 software [24]. A maximum-likelihood (ML) tree was constructed with NS1 and Cap amino acid sequences, using MEGA 7 with the Jones–Taylor–Thornton (JTT) model and 1000 bootstrap replicates. The *p*-distance method was used to reconstruct a neighbor joining (NJ) tree with 1000 bootstrap replicates.

The most recent common ancestor (tMRCA) and rates of nucleotide substitutions per site per year were estimated using the Bayesian Markov chain Monte Carlo (MCMC) method within the BEAST package (Version 1.10.4) [25]. A general time reversible (GTR) substitution model with a proportion of gamma and invariant distributed rate heterogeneity (GTR + G + I) with an uncorrelated relaxed lognormal molecular clock was selected. Chain length for the run was 1 × 10^8^ generations, with sampling at every 10,000 generations. After removing the initial 10% of samples as burn-in, only runs with an estimate sample size (ESS) > 200 were accepted, based on the software Tracer (v1.7.1) (Tracer, http://tree.bio.ed.ac.uk/software/tracer/, 4 July 2020). A maximum clade credibility (MCC) tree was reconstructed with TreeAnnotator (v1.10.4) and displayed in Figtree (v1.4.4) (Figtree, http://tree.bio.ed.ac.uk/software/figtree/, 4 July 2020).

### 2.4. Selection Pressures Analysis

The detection of selected NS1 and Cap coding sequences of PPV7 was performed using DATAMONKEY (Datamonkey, http://www.datamonkey.org/, 4 July 2020). Positive selected sites were detected using 4 algorithms, including fixed effects likelihood (FEL), single-likelihood ancestor counting (SLAC), fast unconstrained Bayesian approximation (FUBAR) and mixed effects model of evolution (MEME) [26,27,28]. A site was considered as a positive selection position only if it was identified by at least 2 algorithms, and with *p* < 0.1 in SLAC, *p* < 0.05 in FEL and MEME and posterior probability > 0.9 in FUBAR were considered significant. The selection pressure analysis of genomes was determined with MEGA7 software by calculating the differences between non-synonymous (dN) and synonymous substitution (dS) rates, for the aligned genes. The calculated value of dN-dS was used to evaluate selection pressure (dN-dS > 0: positive selection; dN-dS < 0: purifying selection; and dN-dS = 0: neutral selection) [29].

### 2.5. Structural Analysis

The physicochemical properties of PPV7 Cap were assessed with the ProtParam webserver (ProtParam, https://web.expasy.org/protparam/, 4 July 2020). The secondary structure of PPV7 Cap was predicted with PSIPRED [30]. 

### 2.6. B Cell Epitope Prediction

Linear B cell epitopes of the PPV7 Cap were predicted using the BepiPred 2.0 online tool in IEDB (Immune Epitope Database) (BepiPred 2.0, http://www.iedb.org/, 4 July 2020), with a threshold of 0.55 (corresponding specificity > 0.817 and sensitivity < 0.292); only epitopes with > 7 residues were considered for subsequent antigenicity analysis. Antigenicity testing was done with the VaxiJen v2.0 server online tool [31] (VaxiJen v2.0, http://www.ddg-pharmfac.net/vaxijen/VaxiJen/VaxiJen.html, 4 July 2020).

### 2.7. CD8 T Cell Epitope Prediction

For CD8 T cell epitopes prediction, 3 porcine MHC-I molecules SLA-1*04:01, SLA-2*04:01 and SLA-3*04:01 [32,33,34] were selected, based on the IEDB recommended 2.22 algorithm in IEDB, with a peptide size of 9 residues. Higher scoring peptides (rank ≤ 2%) (threshold 0.5% and 2.0% rank for strong and weak binders, respectively) based on the prediction were chosen for subsequent immunogenicity evaluation by the VaxiJen v2.0 server.

### 2.8. Peptide Modelling and Molecular Docking

The 3D structures of all peptides were modelled with the PEP-FOLD3 online server [35]. All peptides were docked to correspond to SLA-2*04:02 (PDB ID: 6A6H) and SLA-3*04:01 (PDB ID: 5H94), using the PatchDock rigid-body docking server, using the defined threshold [36]. Based on geometry docking algorithm in PatchDock, the docking transformation with good molecular shape complementarity was selected, and docked complexes were refined with the FireDock server [37,38]. Complexes with high global energy, attractive Vander Waal (vdW) energy and hydrogen bonding energy were used for subsequent analyses. Protein peptide connections were examined with the LigPlus tool and analyzed with Pymol.

## 3. Results

### 3.1. Construction of Phylogenetic Tree and Evolution Dynamic Analysis

**Figure 1:**
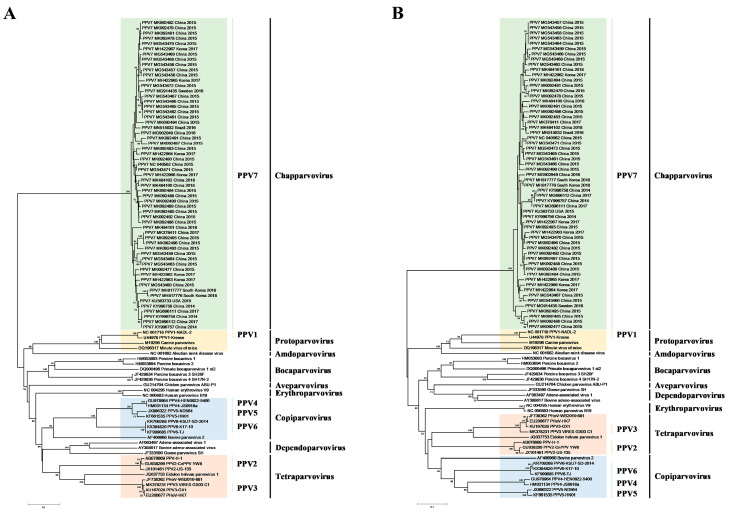
Phylogenetic analysis of viruses in the *Parvoviridae* family. Trees were constructed based on the NS1 (**A**) and Cap (**B**) amino acid sequences by the neighbor joining (NJ) phylogenetic method, using the p-distance model with 1000 bootstrap replicates and bootstrap > 50%.

**Figure 2:**
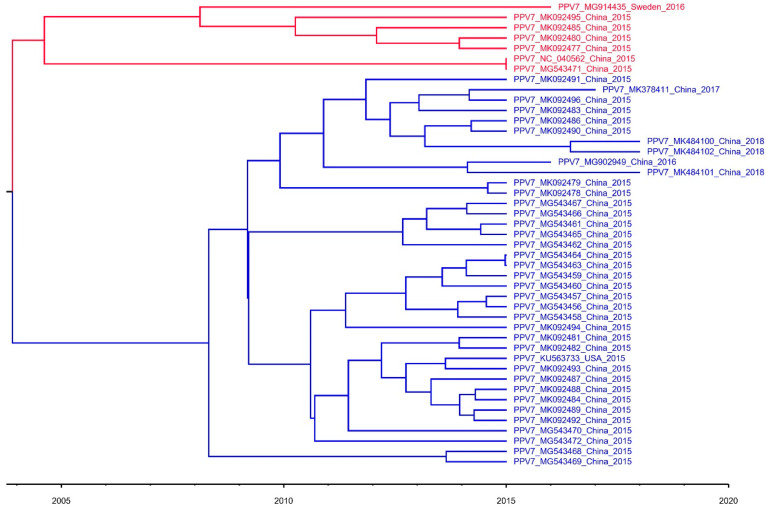
Maximum clade credibility (MCC) tree for the complete genome sequences of PPV7. The tree contained 2 major clades: Clade 1 (red) and Clade 2 (blue).

To better understand genetic relationships between PPV7 and other strains of *Parvoviridae*, a phylogenetic tree was constructed, using the maximum likelihood (ML) and neighbor joining (NJ) methods, based on the NS1 and Cap amino acid sequences, respectively. According to phylogenetic analyses, all PPV7 strains were located in a branch belonging to the *Chapparvovirus* genus (Figure 1 and Figure S1). Additionally, PPV7 was more genetically close to PPV1, compared to the PPV strains among the other genotypes (Figure 1). Furthermore, the maximum clade credibility (MCC) tree was reconstructed with 45 complete PPV7 genomes. It appeared that PPV7 strains may have had a common ancestor in 2004 (95% highest posterior density (HPD): 1986–2014); furthermore, the PPV7 strains from China were the most likely ancestral strains, based on currently available sequences (Figure 2).

The evolutionary rates of the *NS1* and *Cap* genes of PPV7 were estimated. The mean evolutionary rates of the *NS1* and *Cap* genes were 8.01 × 10^−4^ per site per year (95% HPD: 3.67 × 10^−6^–1.9 × 10^−3^) and 2.19 × 10^−3^ per site per year (95% HPD: 1.28 × 10^−4^–5.06 × 10^−3^), respectively.

### 3.2. Selection Pressures Analysis

Five sites (24, 106, 158, 270 and 446) among Cap were confirmed by all 4 methods to be under positive selection. Furthermore, 2 sites (195 and 441) were also confirmed to be under positive selection in Cap by at least 2 methods with *p* < 0.05 by FEL and MEME, *p* < 0.1 by SLAC, and a posterior probability > 0.9 by FUBAR (Table 1). In addition, 8 NS1 sites (69, 342, 426, 465, 474, 498, 525 and 603) were detected as positive selection positions by all 4 methods, whereas the other 9 sites (27, 83, 376, 500, 547, 549, 550, 579 and 618) were detected as positive selection positions by at least 2 methods (Table 2). The overall mean differences of dN-dS were −4.122 for *Cap* gene and −15.371 for *NS1* gene, indicating that both Cap and NS1 were under purifying selection.

### 3.3. Sequence and Structural Characteristics

**Figure 3:**
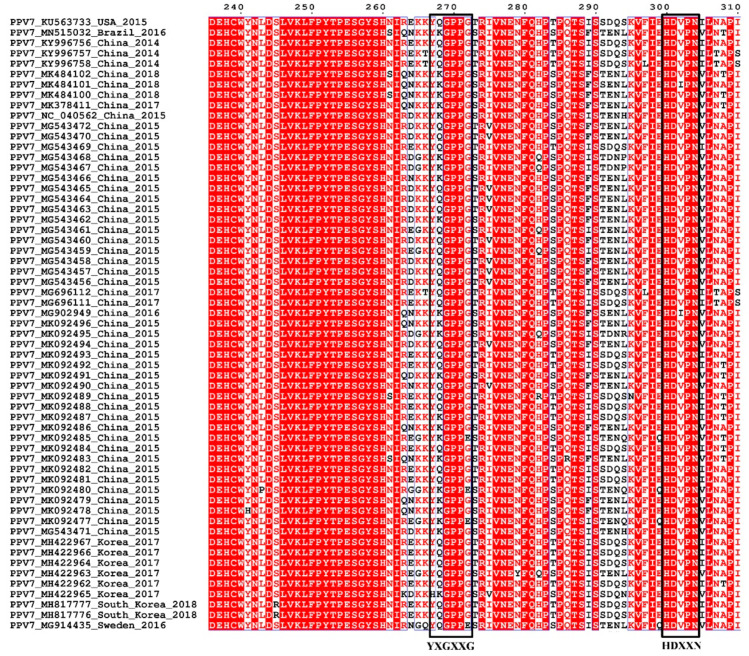
Sequence alignment of Ca^2+^ binding loop and putative secretory phospholipase A2 (PLA2) motif of PPV7. The conserved amino acids of the Ca^2+^ binding loop (^267^YXGXXG^272^) and the catalytic residues (^300^HDXXN^304^) are indicated at the bottom of the alignment.

Sequence alignments revealed that PPV7 genomes (total 45 of isolates deposited in GenBank) exhibited 91.9–100% nucleotide homology. Furthermore, PPV7 *NS1* and *Cap* genes (total 59 of each deposited in GenBank) had 92.8–100% and 85.7–100% homology, respectively, and the derived amino acid sequence of both proteins shared 89.9–100% and 82.4–100% identity, respectively. The Ca^2+^ binding loop (YXGXG) was present in the Caps of PPV1, PPV2, PPV3 and PPV5 [1,10], and “YXGXR” was in PPV6 [11], but absent in PPV4. However, the potential Ca^2+^ binding loop was the ^267^YXGXXG^272^ motif in PPV7, and “^269^GXX^271^” (“^269^GPP^271^”) were strictly conserved in PPV7 (Figure 3). Furthermore, compared to the catalytic motif (HDXXY) of the putative secretory phospholipase A2 (PLA2) in PPV5 [10], a similar motif ^300^HDXXN^304^ was present in PPV7, whereas a point mutation occurred at position 304 (N 304 Y) in the 59 of PPV7 Cap proteins (Figure 3).

A representative strain (KU563733) was used for a structural analysis of PPV7 Cap. The physicochemical properties of the PPV7 Cap, computed using the ProtParam tool, concluded that it contained 469 aa, with a molecular weight of 54,257.8 Da. The isoelectric point (PI) of this protein was 7.23, indicating that it is positive in nature. Out of 469 residues, 42 (Asp + Glu) were negatively charged and 42 (Arg + Lys) were positively charged. The instability index of (II) was computed to be 38.58, indicating that it was a stable protein. The predicted aliphatic index was 56.78 and the grand average of hydropathicity (GRAVY) for the protein sequence was −0.695. This protein contained 7483 atoms, and was described by the formula C2469H3639N647O714S14. The estimated half-life was 30 h (mammalian reticulocytes, in vitro), > 20 h (yeast, in vivo) and > 10 h (*Escherichia coli*, in vivo).

The secondary structure of the PPV7 Cap was analyzed by PSIPRED with (24%) beta sheets, (12%) helixes and (64%) loops present in the structure (Figure S2). VaxiJen v2.0 was used to evaluate the antigenicity of the Cap. By setting the threshold at 0.4 for higher specificity, antigenicity score was 0.4426 for the Cap, implying an excellent antigenic potential.

### 3.4. Linear B Cell Epitopes Prediction and Analysis of Cap

In total, 10 potential linear B-cell epitopes were predicted with BepiPred 2.0, 6 of which were chosen for subsequent analyses based on antigenicity scores evaluated by VaxiJen v2.0 (Table 3). The prediction of the secondary structure and sequence alignment of PPV7 Caps suggested that amino acid mutations occurred predominantly in loops, and the six potential epitopes were all located in these loops (Figure S3). Among these 6 epitopes, epitopes C and E were highly conserved (with 98.3 and 100% identity, respectively) throughout all 59 isolates (Table 3 and Figure S3). The epitope C sequences (IQELRPGKN) in 58 isolates were identical, except that 2 mutations occurred in one isolate of PPV7-77 (IQELMPRKN). However, these 2 mutations in this isolate significantly decreased the antigenic score from 0.7908 to 0.2766. The other 4 epitopes exhibited high variations among these 59 PPV7 isolates, with 1 positive selection site (270K) and 2 sites (441K and 446P) present in epitopes D and F, respectively. Generally, variations of the capsid surface, caused by residue mutations, may alter antigenic profiles, which results in the differences in cross-protective activities. Thus, the alteration of the PPV7 capsid antigenic profile is one of the strategies of PPV7 to adapt to the host during the virus evolution.

**Figure 4:**
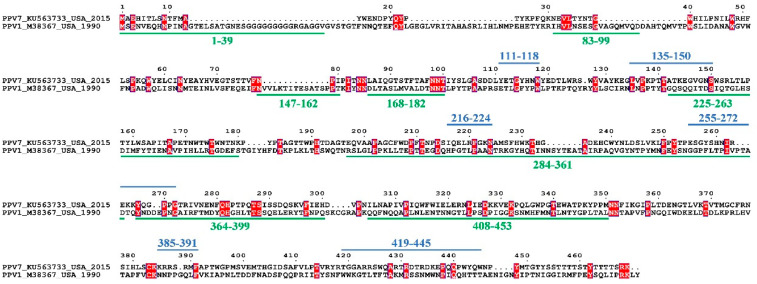
Sequence alignment of predicted PPV7 and identified PPV1 linear B cell epitopes from Cap. Blue lines represent predicted PPV7 B cell epitopes and green lines represent identified PPV1 B cell epitopes.

Further, we reviewed 8 linear B cell epitope regions of PPV1 Cap deposited in IEDB database, according to the corresponding peptide overlap (Table S3). Except for regions A and D, 6 of the 8 linear B cell epitope regions were mapped to the most exposed surface regions of the PPV1 capsid (Figure S4A). Comparison of the epitope sequences revealed that there were no homologous sequences between the PPV7 and PPV1 (Figure 4) due to the low identity (~11.6%) of the Cap, suggesting that there may be a lack of cross-reactivity between the two viruses.

### 3.5. CD8 T Cell Epitopes Prediction and Interaction Study of Predicted Peptides with SLA Alleles

**Figure 5:**
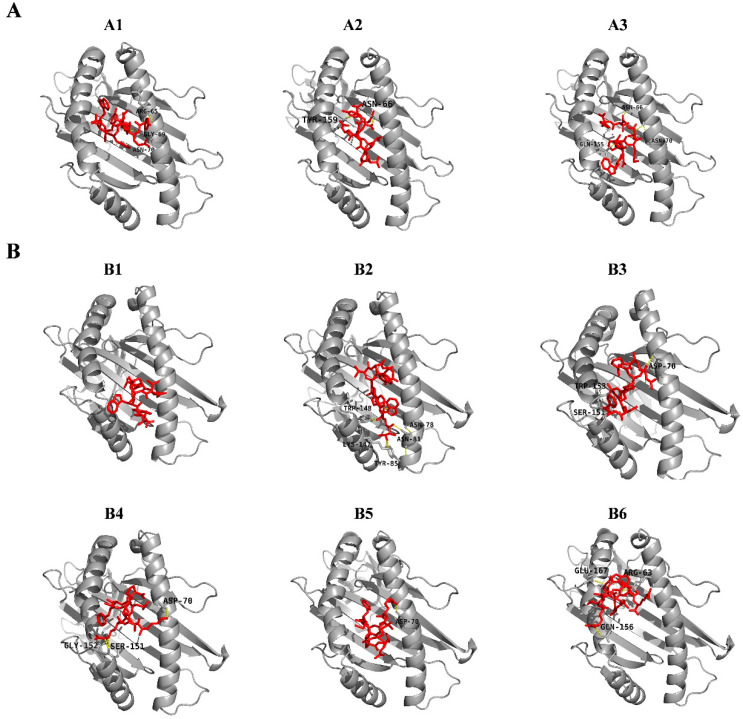
Molecular interaction analysis of PPV7 peptides (shown as red) to SLA-2*0402 (**A**) and SLA-3*0401 (**B**) protein (shown as gray). Interacting residues are shown and are consistent with the information in Table 4.

A total of 12 peptides from the Cap were predicted as CD8 T cell epitopes, whose antigenicities were evaluated with a VaxiJen v2.0 sever (Table S4). The 3D structures of all MHC class-I peptides were modelled via PEP-FOLD3, and a best model for each peptide was used for the subsequent molecular docking with SLA proteins. Among all 12 peptides, 3 peptides were docked to MHC class-I SLA-2*04:02, whereas 6 peptides were docked to MHC class-I SLA-3*04:01. All 9 peptides had high binding affinities. For the 3 peptide-SLA-2*04:02 molecular docking, the binding efficiency of each epitope was evaluated by the global and vdW energies, computed to range from −23.20 to −54.08 kcal/mol and −22.21 to −30.77 kcal/mol, respectively (Table 4). All 3 peptides were predicted to be able to be docked into the groove of the SLA-2*04:02 molecule and form stable hydrogen bonds with the residues (within 3.1Å) in the groove of the SLA. Furthermore, Asn66 and Asn70 residues from the SLA groove were most abundantly involved in bonding with various peptides (Figure 5A and Figure S5A). In addition, global and vdW energies of the 6 peptide-SLA-3*04:01 dockings ranged from −17.93 to −44.82 kcal/mol and −18.96 to −26.27 kcal/mol, respectively (Table 4). Of these 6 peptides, hydrogen bonds <3Å were frequently observed in 5 docking complexes to form stable complexes (Figure 5B and Figure S5B). However, the peptide (TAPETNWTW) docked into the groove of SLA-3*04:01 and non-formed hydrogen bonds, whereas the major hydrophobic component interacted with SLA. Notably, 2 overlapped peptides (KRRSRMFAP and RRSRMFAPT) had higher immunogenicity scores (1.2953 and 0.9148), and were strictly conserved in all 59 PPV7 Cap sequences (Table 4).

## 4. Discussion

Since the discovery of PPV7 in 2016, most studies have focused on the genetic characterization of individual isolates and epidemiological investigations [13,14,15,16,17]. However, the origin and evolution of this newly emerging PPV are also of interest. To better understand the evolution and genetic relationships of various PPV7 strains, we constructed an MCC tree based on the complete genome sequences of PPV7. We have determined that PPV7 strains have two major clades and may have a common ancestor in approximatively 2004. Furthermore, the PPV7 strains isolated from China are the most likely ancestral strains, based on the collected sequences (Figure 2). To better define PPV7 genotypes, we constructed the NJ and ML trees using NS1 and Cap, respectively, but they did not display similar clusters (data not shown). Thus, a phylogenetic tree analysis did not provide strong evidence for PPV7 genotyping based on current sequences.

The mean evolutionary rates of the PPV7 *NS1* gene rate (8.01 × 10^−4^ per site per year) were higher than that of the PPV1 *NS1* gene (3.03 × 10^−5^ per site per year) from the previous report [39]. In the PPV7 genotype, the *Cap* gene had a more rapid evolutionary rate (2.19 × 10^−3^ per site per year) than the *NS1* gene (8.01 × 10^−4^ per site per year), which was comparable to the rates of most RNA viruses [40]. In addition, the evolutionary rate of the PPV7 *Cap* gene was also higher than the rates of the PPV1, PPV2, PPV3 and PPV4 *Cap* genes (10^−4^ per site per year) [39,41]. We also analyzed selective pressures of the PPV7 *Cap* and *NS1* genes. The overall mean difference of dN-dS was −4.122 for the *Cap* gene, which was higher than that of the *NS1* gene (−15.371). Therefore, we inferred that both the *Cap* and *NS1* genes are under purifying selection, whereas the *Cap* gene undergoes more stringent purifying selection than the *NS1* gene. This was supported by the higher evolutionary rate of Cap compared to NS1, and further supported the notion of the correlation between selective pressure and evolutionary rate. PPV7 employs the antigenic variations/shift to resist selective pressures from the host’s immune system in the absence of vaccine-induced immune pressure.

The PPV Cap is considered a primary target for eliciting neutralizing antibodies, and has been used for the key antigen of the subunit vaccine against PPV [20,21]. With virtually no information regarding the immune responses against PPV7, computer-based immune-informatics can be used for analysis of antigenic profiles and assist in vaccine development against this newly emerging virus, thereby decreasing cost and time. By selecting the effective antigenic components (epitopes) exposed on the surface, epitope-based vaccines have great potential, as they are capable of inducing strong immune responses in hosts. In this study, we explored potential B cell and CD8 T cell epitopes of PPV7 Cap that may elicit immune responses in the host. After filtering, a total of six linear B cell epitopes were predicted in PPV7 Cap located in loops (Figure S3). However, four of the six epitopes were highly variable in residue compositions (Figure S3), although it was not determined whether these epitopes were located on the capsid surface. We tried to predict the 3D structure of PPV7 Cap, but failed due to low identity (~11.6%) with the template of PPV1 Cap (data not shown). The loops of the PPV1 Cap are generally located on the capsid surface, and these loops were the dominant B cell epitope regions and were considered to be important for viral infection and immunogenicity (Figure S4). Amino acid mutations occurred predominantly in these loops [42], and as PPV1 Cap loops are generally located on the capsid surface, these amino acid mutations may influence receptor binding or antigenicity. Thus, high variations of the epitopes located in PPV7 Cap loops may influence receptor binding or antigenicity, if these epitopes are mostly located on the capsid surface; therefore, perhaps PPV7 uses antigenic shift to escape host immune responses. Interestingly, epitope C (IQELMPRKN to IQELRPGKN) had two point mutations (see underlined residues) in isolate PPV7-77, which dramatically decreased the antigenicity score from 0.7908 to 0.2766. Thereafter, the antigenicity of the peptide containing the double-point mutation in isolate PPV7-77 greatly decreased, which may be one of the strategies of this virus to evade the host’s immune responses. Of note, epitope E (KRRSRMF) was highly antigenic (antigenicity score = 1.6044) and strictly conserved among all the isolates of PPV7. Therefore, this potential candidate can be used as a dominant antigen for PPV7 serodiagnosis. Additionally, we predicted several potential CD8 T cell epitopes derived from the PPV7 Cap, and identified nine peptides able be docked onto the SLA with high binding affinities. Based on molecular docking, peptides with potential to SLA and high immunogenicity scores may prove highly immunogenic. Importantly, two conserved overlapped peptides (KRRSRMFAP and RRSRMFAPT) with high immunogenicity scores (1.2953 and 0.9148) should be experimentally tested for the PPV7 vaccine in future studies.

Many parvoviruses, including porcine parvovirus (PPV), human parvovirus B19 (B19V) and human bocavirus 1 (HBoV1), cause infections of their hosts. Among them, B19V is an important human pathogen responsible for a variety of diseases and causes various pathological symptoms, including nonimmune hydrops fetalis and fetal death in pregnant women [43]. Vaccination is the most effective strategy against B19V pathogenesis and infection. Currently, there are two generations of B19V based virus like particles (VLPs) vaccines. The first generation of B19 VLPs consists of two viral structural proteins (VP1 and VP2) produced in the baculovirus expression system, and this induces neutralizing antibodies [44]. These vaccines, produced in insect cells, induced side effects, e.g., reactive symptoms in the host; therefore, baculovirus-based vaccines were abandoned during clinical trials. The second generation of B19 VLPs vaccines, composed of only VP2 protein, are expressed in *Saccharomyces cerevisiae* [44]. Until now, research for vaccines against B19V has been a huge challenge, due to the unavailability of viral antigens, a good cell line model and a virus-infected animal model. Moreover, there is almost no viremia in most B19V-infected patients when symptoms start to appear. Neutralizing antibodies against B19V are insufficient, emphasizing the importance of developing a vaccine that induces innate and cellular immunity against B19V. The research work of the B19V vaccine provides a further design reference for the PPV7 vaccine.

Based on accumulated evidence, this newly emerging PPV7 has epidemic potential in the global swine population. There is limited information about its epidemiology, transmission, pathogenesis and molecular biology, or indeed, how PPV7 emerges in swine. Regardless, its presence in aborted pig fetuses and its co-infection with PCV2 imply that PPV7 threatens swine herd health security. Notably, the substitution rate of PPV7 was higher than PPV1-4, which may enable PPV7 to adapt to various environmental conditions and cause a substantial threat to the swine herd. Thus, a vaccine against PPV7 is needed to control this emerging virus infection. Inactivated vaccines against PPV1 have been used for 30 years because they hindered or reduced virus transmission. Low homology between PPV7 Cap and PPV1 suggested the PPV1 vaccine strains were not closely matched with PPV7 strains. Novel vaccine formulations containing PPV1 and the newly circulating strains PPV7 may overcome some potential weaknesses of current vaccines, perhaps increasing vaccine efficacy.

## 5. Conclusions

In conclusion, this study has provided evidence on the evolutionary dynamics of PPV7. We concluded that PPV7 has a more rapid evolutionary rate than other PPV genotypes. In addition, antigenic profiles of the PPV7 Cap were revealed by immunoinformatics, and there were indications that PPV7 evades the host’s immune responses via antigenic shifts during virus evolution, in the absence of vaccine-induced immune pressure. In addition, these potential B cell epitopes identified in this study may serve as antigens for PPV7 vaccine or for serological diagnosis, with further experiments warranted.

## Figures and Tables

**Table 1:** Selection analysis of PPV7 Cap coding sequences.

FEL			SLAC			FUBAR			MEME		
Site	dN-dS	*p*-Value	Site	dN-dS	*p*-Value	Site	dN-dS	Post.Pro	Site	β^+^	*p*-Value
24	2.492	0.004	24	5.741	0.018	24	6.900	0.997	24	2.5	0.01
106	2.157	0.023	106	4.943	0.028	106	5.706	0.979	106	24.21	0
158	1.552	0.033	158	3.652	0.098	158	3.244	0.972	158	1.55	0.05
						195	1.442	0.909	195	166.2	0
270	1.777	0.026	270	5.107	0.048	270	4.363	0.974	270	14.87	0
						441	2.926	0.944	441	18.72	0
446	1.649	0.015	446	3.970	0.055	446	3.901	0.988	446	3.43	0.02

**Table 2:** Selection analysis of PPV7 NS1 coding sequences.

FEL			SLAC			FUBAR			MEME		
Site	dN-dS	*p*-Value	Site	dN-dS	*p*-Value	Site	dN-dS	Post.Pro	Site	β^+^	*p*-Value
27	1.519	0.016				27	2.24	0.977	27	1.52	0.03
69	2.815	0.002	69	3.466	0.069	69	4.953	0.997	69	2.82	0
						83	2.627	0.96	83	148.66	0
342	5.477	0.01	342	8.331	0.005	342	12.563	0.993	342	173.79	0
			376	4.566	0.066	376	5.16	0.965			
426	8.215	0.005	426	10.973	0.002	426	17.289	0.999	426	40.1	0
465	1.905	0.034	465	3.658	0.088	465	2.882	0.979	465	1.9	0.05
474	4.118	0.019	474	5.486	0.026	474	7.406	0.983	474	4.12	0.03
498	4.261	0	498	5.326	0.031	498	8.12	1	498	4.25	0
			500	5.817	0.028	500	2.963	0.956			
525	7.599	0.003	525	10.047	0.002	525	15.702	0.999	525	7.6	0
			547	5.486	0.026	547	4.412	0.97			
			549	4.267	0.059	549	2.533	0.956			
			550	3.658	0.088	550	2.944	0.992	550	1.9	0.01
579	2.081	0.01				579	3.455	0.994	579	2.08	0.02
603	1.271	0.025	603	3.778	0.081	603	1.998	0.974	603	1.27	0.04
			618	4.105	0.077	618	2.663	0.95			

**Table 3:** Linear B cell epitopes prediction of PPV7 Cap using BepiPred 2.0.

Epitope	Start	End	Sequence	Length	Identity (59 Isolates)	Vaxijen v2.0 Score
A	111	118	YETGYHNW	8	72.88%	0.5888
B	135	150	LVPKPTTATKEGVGNS	16	18.64%	0.4355
C	216	224	IQELRPGKN	9	98.31%	0.7908
D	255	272	ESGYSHNIREKKYQGPPG	18	25.42%	0.6235
E	385	391	KRRSRMF	7	100.00%	1.6044
F	419	445	TGGARRSWQARTRDTRDKEPQQPWYQW	27	10.17%	0.4966

**Table 4:** Molecular docking results of SLA-2*0402 and SLA-3*0401 with MHC I peptides.

No.	Allele	Start	End	Peptide	Global Energy (kcal/mol)	vdW Energy (kcal/mol)	H-Bond Energy (kcal/mol)	Interacting Residues	Vaxijen Score	Identity (59 Isolates)
A1	SLA-2*0402	111	119	YETGYHNWY	−26.32	−22.21	−1.43	Arg65, Gly69, Asn70	0.5659	72.88%
A2	SLA-2*0402	409	417	AFVLPTVRY	−23.20	−24.38	−1.49	Asn66, Tyr159	0.7382	91.53%
A3	SLA-2*0402	445	453	WNPYMTGTY	−54.08	−30.77	−3.19	Asn66, Asn70, Gln155	1.2177	32.20%
B1	SLA-3*0401	166	174	TAPETNWTW	−25.49	−21.17	−0.82		1.4351	96.61%
B2	SLA-3*0401	170	178	TNWTWTWNT	−37.42	−22.35	−4.1	Asn78, Asn81, Tyr85, Lys147, Trp148	1.2242	33.90%
B3	SLA-3*0401	220	228	RPGKNAMSF	−44.82	−24.06	−1.49	Asp70, Ser151, Trp153	0.5339	91.53%
B4	SLA-3*0401	385	393	KRRSRMFAP	−33.28	−26.27	−3.37	Asp70, Ser151, Gly152	1.2953	100.00%
B5	SLA-3*0401	386	394	RRSRMFAPT	−32.49	−18.96	−0.51	Asp70	0.9148	100.00%
B6	SLA-3*0401	423	431	RRSWQARTR	−17.93	−20.24	−2.17	Arg63, Gln156, Glu167	0.6504	76.27%

## Supplementary Materials

The following are available online at https://www.mdpi.com/2076-393X/8/3/359/s1, Figure S1: Phylogenetic analysis of viruses in the *Parvoviridae* family, Figure S2: Secondary structure analysis of PPV7 Cap via PSIPRED, Figure S3: Sequence alignment of PPV7 Cap, Figure S4: Mapping the location of identified PPV1 B cell epitope regions on 3D structure (PDB ID: 1K3V) of capsid (A) and Cap monomer (B,C), Figure S5: 2D graphical representation of molecular interaction analysis of MHC class-I alleles binding peptides to SLA-2*0402 (A) and SLA-3*0401 (B) protein, Table S1: Detailed information of PPV7 strain sequences used in this study, Table S2: Detailed information of reference *Parvoviridae* sequences used in this study, Table S3: Identified linear B cell epitopes in PPV1 Cap deposited in IEDB database, Table S4: CD8 T cell epitopes prediction of PPV7 Cap.
