# Supplementary material for: Porcine Parvovirus 7: Evolutionary Dynamics and Identification of Epitopes toward Vaccine Design
Source: Vaccines (Basel). 2020 Jul 5;8(3):359. doi: 10.3390/vaccines8030359 (PMC7565409; doi:10.3390/vaccines8030359)
Supplement: Supplementary file 1 [file vaccines-08-00359-s001.pdf]

Table S1. Detailed information of PPV7 strain sequences used in this study.

GenBank Number	Strain Name	Country	Year	Host	Genome	NS1	Cap
MK484102	PPV7/China/AHhf	China	2018		3999 bp	2019 bp	1425 bp
MK484101	PPV7/China/AHmas	China	2018		3999 bp	2019 bp	1425 bp
MK484100	PPV7/China/AHbz	China	2018		3999 bp	2019 bp	1425 bp
MK378411	PPV7_VIRES_GZ02_C1	China	2017	Sus scrofa	4036 bp	2019 bp	1425 bp
MH422967	PPV7-KF6	Korea	2017		3674 bp	2019 bp	1410 bp
MH422966	PPV7-KF5	Korea	2017		3656 bp	2019 bp	1392 bp
MH422965	PPV7-KF4	Korea	2017		3689 bp	2019 bp	1425 bp
MH422964	PPV7-KF3	Korea	2017		3668 bp	2019 bp	1404 bp
MH422963	PPV7-KF2	Korea	2017		3674 bp	2019 bp	1410 bp
MH422962	PPV7-KF1	Korea	2017		3689 bp	2019 bp	1425 bp
MG914435	PPV7_SWE20	Sweden	2016		4062 bp	2019 bp	1401 bp
MG543471	GX49	China	2015		3999 bp	2019 bp	1425 bp
MG543472	GX50	China	2015		3999 bp	2019 bp	1425 bp
MG543470	GX47	China	2015		3984 bp	2019 bp	1410 bp
MG543469	GX48	China	2015		3999 bp	2019 bp	1425 bp
MG543468	GX45	China	2015		3999 bp	2019 bp	1425 bp
MG543467	GX44	China	2015		3987 bp	2019 bp	1413 bp
MG543466	GX35	China	2015		3999 bp	2019 bp	1425 bp
MG543465	GX34	China	2015		3999 bp	2019 bp	1425 bp
MG543464	GX32	China	2015		3999 bp	2019 bp	1425 bp
MG543463	GX31	China	2015		3999 bp	2019 bp	1425 bp
MG543462	GX30	China	2015		3999 bp	2019 bp	1425 bp
MG543461	GX29	China	2015		3999 bp	2019 bp	1425 bp
MG543460	GX28	China	2015		3987 bp	2019 bp	1413 bp
MG543459	GX6	China	2015		3999 bp	2019 bp	1425 bp
MG543458	GX5	China	2015		3999 bp	2019 bp	1425 bp
MG543457	GX3	China	2015		3999 bp	2019 bp	1425 bp
MG543456	GX2	China	2015		3999 bp	2019 bp	1425 bp
MG696112	FJLY2017	China	2017		3588 bp	2019 bp	1401 bp
MG696111	FJFZ2017	China	2017		3588 bp	2019 bp	1401 bp
KU563733	42	USA	2015		4103 bp	2019 bp	1410 bp
KY996756	GD-2014-1	China	2014		3443 bp	2019 bp	1410 bp
KY996757	GD-2014-2	China	2014		3437 bp	2019 bp	1401 bp
KY996758	GD-2014-3	China	2014		3437 bp	2019 bp	1401 bp
MN515032	BR_RSPPV7	Brazil	2016		3727 bp	2019 bp	1425 bp
MG902949	37	China	2016	tonsil	4013 bp	2019 bp	1425 bp
MK092496	PPV7-JX38	China	2015	wild boar	3981 bp	2019 bp	1410 bp
MK092495	PPV7-JX21	China	2015	wild boar	3973 bp	2019 bp	1401 bp
MK092494	PPV7-JX15	China	2015	wild boar	3997 bp	2019 bp	1425 bp
MK092493	PPV7-JX10	China	2015	wild boar	3982 bp	2019 bp	1401 bp
MK092492	PPV7-DJH26	China	2015	wild boar	3982 bp	2019 bp	1410 bp
MK092491	PPV7-DJH24	China	2015	wild boar	3997 bp	2019 bp	1425 bp
MK092490	PPV7-DJH23	China	2015	wild boar	3997 bp	2019 bp	1425 bp
MK092489	PPV7-DJH20	China	2015	wild boar	3982 bp	2019 bp	1410 bp
MK092488	PPV7-DJH19	China	2015	wild boar	3982 bp	2019 bp	1410 bp
MK092487	PPV7-DJH14	China	2015	wild boar	3982 bp	2019 bp	1410 bp
MK092486	PPV7-DJH13	China	2015	wild boar	3994 bp	2019 bp	1422 bp

MK092485	PPV7-DJH12	China	2015	wild boar	3973 bp	2019 bp	1401 bp
MK092484	PPV7-DJH11	China	2015	wild boar	3979 bp	2016 bp	1410 bp
MK092483	PPV7-LB4	China	2015	wild boar	3994 bp	2019 bp	1422 bp
MK092482	PPV7-87-clone2	China	2015	wild boar	3982 bp	2019 bp	1410 bp
MK092481	PPV7-87-clone1	China	2015	wild boar	3997 bp	2019 bp	1425 bp
MK092480	PPV7-80	China	2015	wild boar	3973 bp	2019 bp	1401 bp
MK092479	PPV7-77	China	2015	wild boar	3997 bp	2019 bp	1425 bp
MK092478	PPV7-60	China	2015	wild boar	3997 bp	2019 bp	1425 bp
MK092477	PPV7-55	China	2015	wild boar	3973 bp	2019 bp	1401 bp
NC_040562	GX49	China	2015		3999 bp	2019 bp	1425 bp
MH817777	N133	South Korea	2018		3446 bp	2019 bp	1410 bp
MH817776	N141	South Korea	2018		3446 bp	2019 bp	1410 bp

Table S2. Detailed information of reference *Parvoviridae* sequences used in this study.

Sub-family Parvovirinae (genus)	Genbank Number	Strain Name	Country	Host	Year
Tetraparvovirus	JF738362	PHoV-WB2010-681	Romania	Wild boar	2010
	KU167028	PPV3-GX1	China	Swine	
	MK378231	PPV3_VIRES_GX03_C1	China	Sus scrofa	2017
	EU200677	PHoV-HK7	China		
	JQ037753	Eidolon helvum parvovirus 1	Ghana	Eidolon helvum	
	AB076669	PPV2-H-1	Myanmar	Swine	
	GU938299	PPV2-CnPPV_YW8	China	Swine	2009
	JX101461	PPV2-US-135	USA	Pig	2011
Copiparvovirus	AF406966	Bovine parvovirus 2			
	KR709268	PPV6-KSU7-SD-2014	USA	Swine	2014
	KX384820	PPV6-K17-10	Poland	Swine	
	KF999685	PPV6-TJ	China	Swine	2012
	GU978964	PPV4-HEN0922-5400	China	Swine	2009
	HM031134	PPV4-JS0918a	China	Swine	2009
	JX896322	PPV5-ND564	USA	Swine	2011
	KF661535	PPV5-HN01	China	Swine	2013
Erythroparvovirus	NC_004295	Human erythrovirus V9		Homo sapiens	
	NC_000883	Human parvovirus B19		Homo sapiens	
Dependoparvovirus	AF063497	Adeno-associated virus 1			
	JF333590	Goose parvovirus SH	China	Anser anser	2009
	AY388617	Bovine adeno-associated virus			
Aveparvovirus	GU214704	Chicken parvovirus ABU-P1	Hungary		
Bocaparvovirus	JF429834	Porcine bocavirus 3 SH20F	Hong Kong	Pig	2007
	JF429836	Porcine bocavirus 4 SH17N-2	Hong Kong	Pig	2007
	DQ000496	Primate bocaparvovirus 1 st2			
	HM053693	Porcine bocavirus 1	China	Pig	2006
	HM053694	Porcine bocavirus 2	China	Pig	2006
Amdoparvovirus	NC_001662	Aleutian mink disease virus			
Protoparvovirus	DQ196317	Minute virus of mice			
	M19296	Canine parvovirus		Norden dog kidney cell	

NC_001718	PPV1-NADL-2
U44978	PPV1-Kresse

Table S3. Identified linear B cell epitopes in PPV1 Cap deposited in IEDB database.

Epitope ID	Sequence	Start	End	Epitope Region
104132	MSENVEQHNPINAGT	1	15	A (1-39)
104327	VEQHNPINAGTELSAT	5	20	
103886	EQHNPINAGTELSAT	6	20	
104205	QHNPINAGTELSATG	7	21	
103972	HNPINAGTELSATGN	8	22	
104175	PINAGTELSATGNESG	10	25	
103792	AGTELSATGNESGGG	13	27	
104299	TELSATGNESGGGGGGGGGRGAGGV	15	39	
103942	GNESGGGGGGGGG	21	33	
103986	IHVLNSESGSAGQMVQD	83	99	B (83-99)
104161	NVVLKTITESATSPPT	147	162	C (147-162)
103853	DLTASLMVALDTNNT	168	182	D (168-182)
103947	GQSQQITDSIQTGLH	225	239	E (225-263)
461857	QQITDA	228	233	
104036	LHSDIMFYTIENAVPIHLL	238	256	
104369	YTIENAVPIHLLRTGDEFS	245	263	
461874	RSLGLPPK	284	291	F (284-361)
104049	LPPKLLTEPTTEGDQHPGT	288	306	
104057	LTEPTTEGDQHPGTLPAANT	293	312	
104165	PAANTRKGYHQTMNNSYTE	308	326	
104314	TRKGYHQTMNNSY	312	324	
104298	TEATAIRPAQVGYNT	325	339	
104295	TAIRPAQVGYNTPYMNF	328	344	
461776	FEYSNGGPFLTPI	344	356	
104367	YSNGGPFLTPIVPTAD	346	361	
104362	YNDDEPNGAIRFTMG	364	378	G (364-399)
104143	NDDEPNGAIRFTMGYQHGQ	365	383	
103842	DEPNGAIRFTMGYQH	367	381	
104147	NGAIRFTMGYQHGQ	370	383	
104310	TMGYQHGQLTTSS	376	388	
104202	QHGHLTTSSQELERYTFNP	380	398	
104319	TTSSQELERYTFNPQ	385	399	
104213	QQFNQQAPLNLENTNNGTL	408	426	H (408-453)
104051	LPSDPIGGKSNMHFMNTL	427	444	
104152	NMHFMNTLNTYGPLTAL	437	453	

Table S4. CD8 T cell epitopes prediction of PPV7 Cap.

Allele	Start	End	Length	Peptide	Percentile Rank	Vaxijen 2.0 Score
SLA-2*0401	111	119	9	YETGYHNWY	1.76	0.5659
SLA-3*0401	166	174	9	TAPETNWTW	0.31	1.4351
SLA-3*0401	168	176	9	PETNWTWTW	0.74	1.419
SLA-3*0401	170	178	9	TNWTWTWNT	0.28	1.2242
SLA-3*0401	220	228	9	RPGKNAMSF	1.05	0.5339
SLA-3*0401	224	232	9	NAMSFHWKT	1.65	1.5461
SLA-3*0401	384	392	9	KKRRSRMFA	0.31	0.5991
SLA-3*0401	385	393	9	KRRSRMFAP	0.26	1.2953
SLA-3*0401	386	394	9	RRSRMFAPT	1.36	0.9148
SLA-2*0401	409	417	9	AFVLPTVRY	0.85	0.7382
SLA-3*0401	423	431	9	RRSWQARTR	1.1	0.6504
SLA-2*0401	445	453	9	WNPYMTGTY	1.22	1.2177

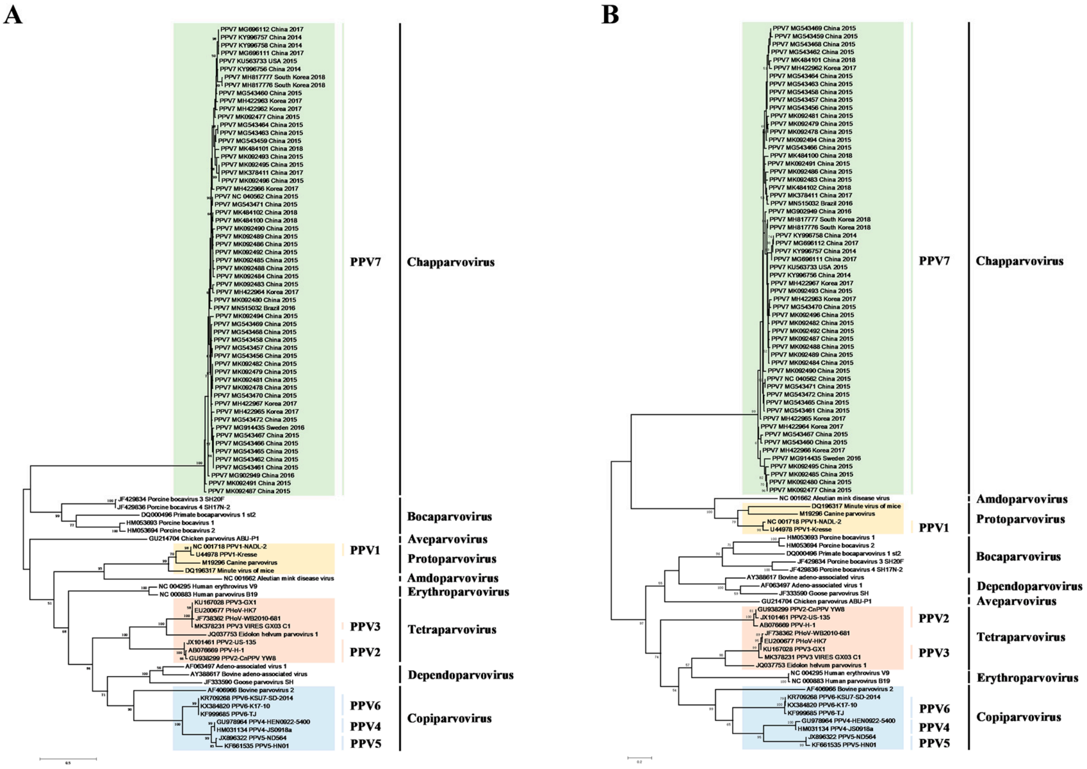

Figure S1. Phylogenetic analysis of viruses in the *Parvoviridae* family. Trees were constructed based on the NS1 (**A**) and Cap (**B**) amino acid sequences by the Maximum-likelihood (ML) phylogenetic method using Jones-Taylor-Thornton (JTT) model with 1000 bootstrap replicates and bootstrap > 50%.

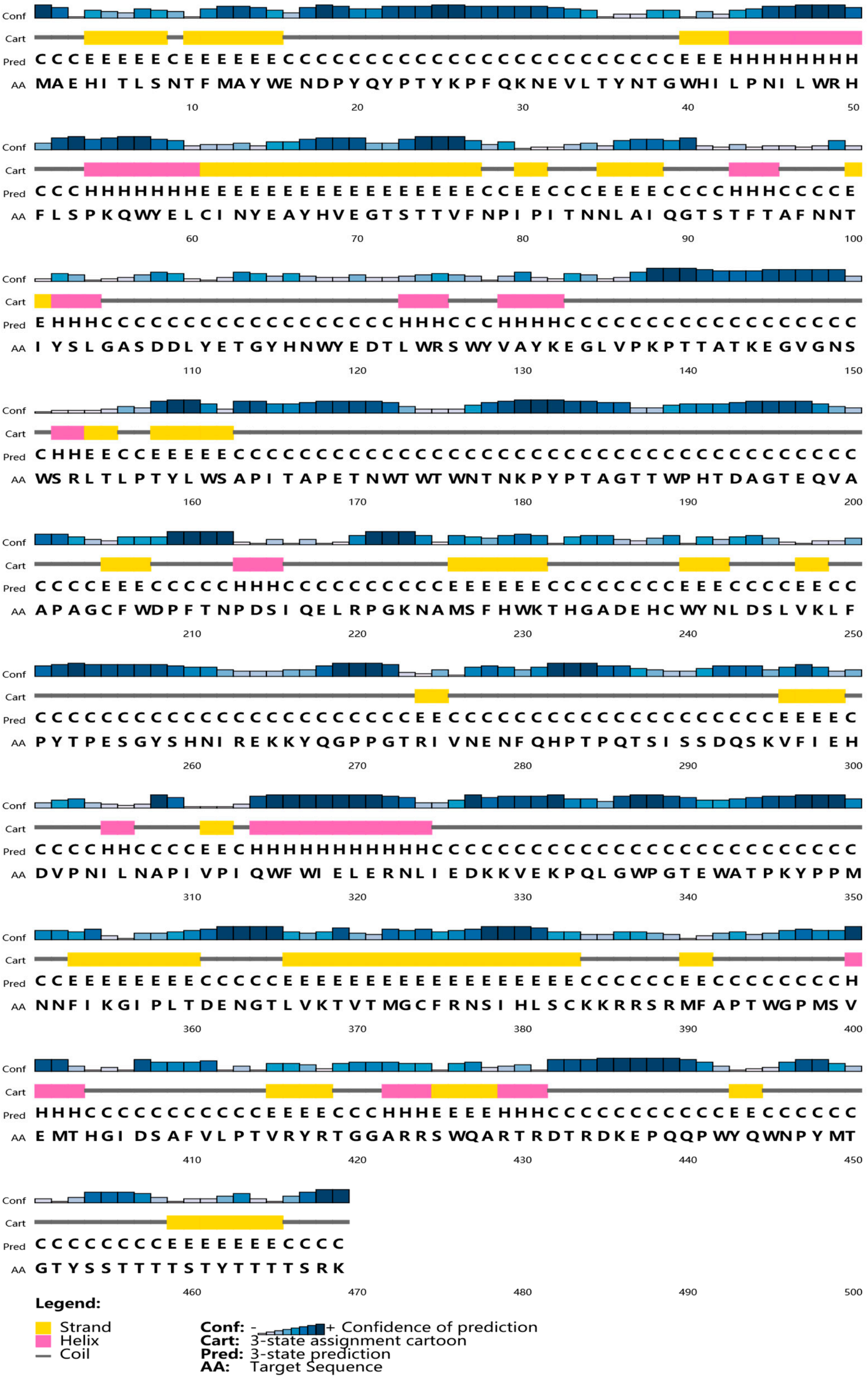

Figure S2. Secondary structure analysis of PPV7 Cap via PSIPRED. Helix, strand and random coil regions are shown as pink, yellow and black, respectively.

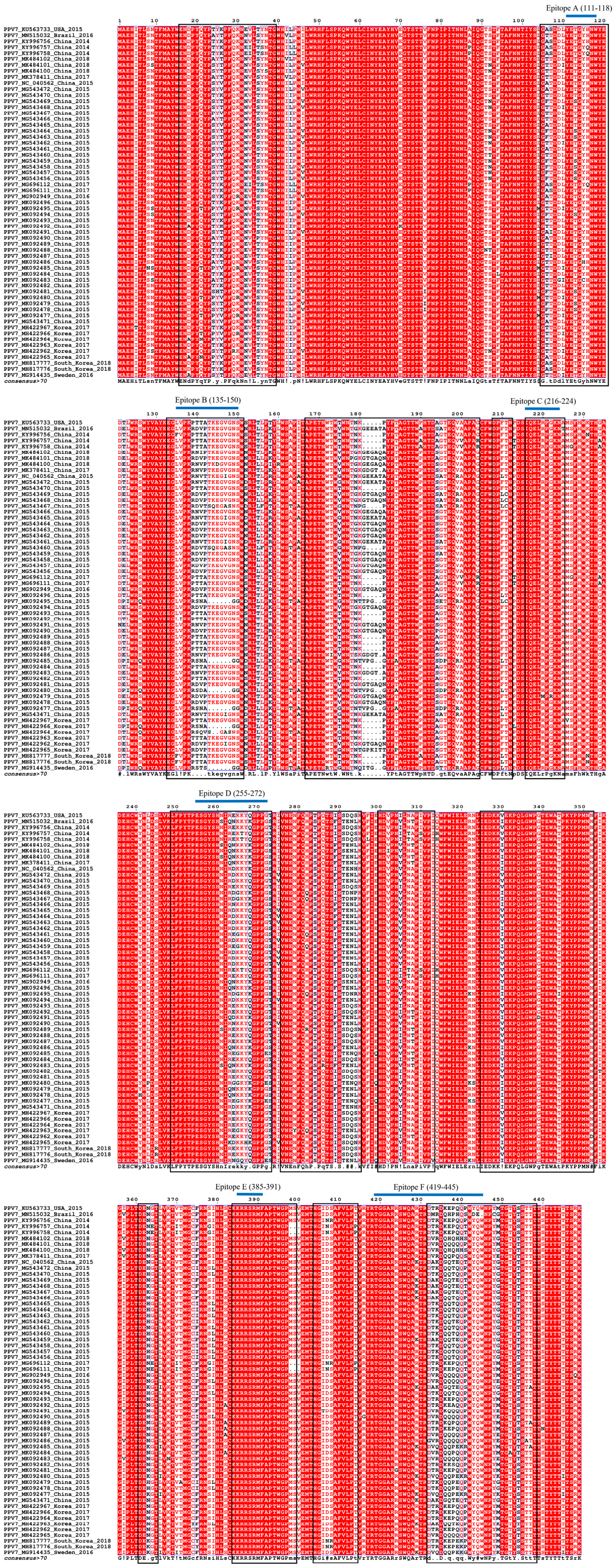

Figure S3. Sequence alignment of PPV7 Cap. All loops were labelled with black rectangles according to the secondary structure prediction. The 6 predicted linear B cell epitopes were labelled with blue lines.

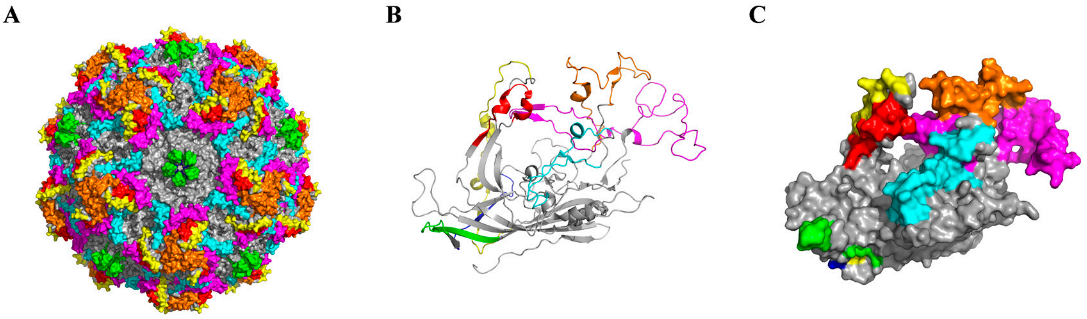

Figure S4. Mapping the location of identified PPV1 B cell epitope regions on 3D structure (PDB ID: 1K3V) of capsid (**A**) and Cap monomer (**B** and **C**). Note: (region B: red, region C: green, region D: blue, region E: yellow, region F: magenta, region G: cyan and region H: orange).

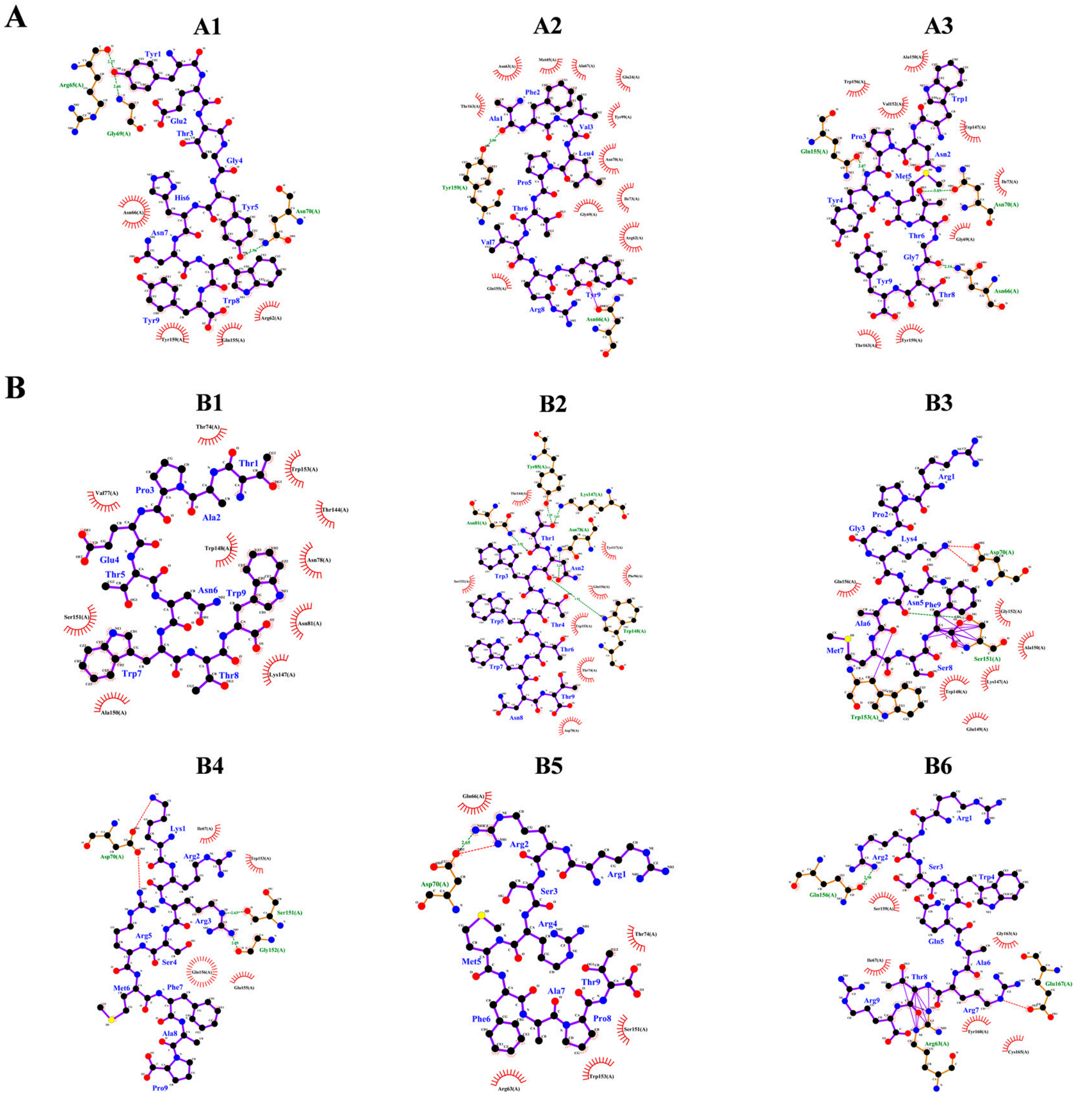

Figure S5. 2D graphical representation of molecular interaction analysis of MHC class-I alleles binding peptides to SLA-2*0402 (**A**) and SLA-3*0401 (**B**) protein. The figure is in symmetry with the information provided in Table 4 and Figure 5 and showing residues interacting with strong hydrogen bonding.
